# Online Conversations for Cognitive Health: A Culturally Adapted Intervention for Older Chinese Immigrants

**DOI:** 10.1093/geroni/igaf122.193

**Published:** 2025-12-31

**Authors:** Kexin Yu, Amanda Mar, Sabrina Lin, Kevin Duff, Lisa Silbert, Hiroko Dodge

**Affiliations:** UT Southwestern Medical Center, Dallas, Texas, United States; Oregon Health & Science University, Portland, Oregon, United States; Lewis & Clark University, Portland, Oregon, United States; Oregon Health & Science University, Portland, Oregon, United States; Oregon Health & Science University, Portland, Oregon, United States; Massachusetts General Hospital, Harvard Medical School, Boston, Massachusetts, United States

## Abstract

Social isolation is a major risk factor for Alzheimer’s Disease and Related Dementias (ADRD), particularly among older Chinese immigrants who face language barriers and social adjustment challenges. To address this, we developed C-CONECT (Chinese Conversation Engagement Co-design and Pilot Test), an adaptation of the I-CONECT study (ClinicalTrials.gov Identifier: NCT02871921, PI: Dodge), which previously demonstrated that user-friendly video chats could improve cognition and social satisfaction in socially isolated older adults. C-CONECT tailored this intervention to the cultural and linguistic needs of older Chinese immigrants. Participants engaged in 30-minute video conversations four times per week over six weeks with trained research staff. Outcomes included loneliness, depressive symptoms, social isolation, and cognitive function, assessed before and after the intervention. We conducted qualitative interviews to understand participant experiences more in-depth. Eight participants (mean age 76, 75% female) enrolled, with seven completing the study. Most (88%) preferred Mandarin, and the chat completion rate was 90%. After the intervention, 62% of participants reported reduced depressive symptoms. Interviews revealed that participants found the conversations warm and supportive, with memory improvement being their primary motivation for participation. High engagement and positive feedback suggest the feasibility and acceptability of C-CONECT in this population. These findings support the potential for a future randomized clinical trial to assess its impact on cognitive function. By culturally adapting evidence-based interventions, we can expand access to effective behavioral health strategies for underrepresented groups, ultimately contributing to ADRD prevention.

